# Heparin-induced thrombocytopenia: a challenging diagnosis in haemodialysis—state of art and review of the literature

**DOI:** 10.1093/ckj/sfag062

**Published:** 2026-02-25

**Authors:** Ivana Capuano, Eleonora Riccio, Pasquale Buonanno, Antonella Tufano, Antonio Pisani

**Affiliations:** Department of Public Health, Chair of Nephrology, University of Naples “Federico II”, Naples, Italy; Department of Public Health, Chair of Nephrology, University of Naples “Federico II”, Naples, Italy; Department of Neurosciences, Reproductive and Odontostomatological Sciences, University of Naples “Federico II”, Naples, Italy; Department of Clinical Medicine and Surgery, Regional Reference Centre for Coagulation Disorders, University of Naples “Federico II”, Naples, Italy; Department of Public Health, Chair of Nephrology, University of Naples “Federico II”, Naples, Italy

**Keywords:** anticoagulation, haemodialysis, heparin, thrombocytopenia, treatment

## Abstract

Heparin-induced thrombocytopenia (HIT) is a life-threatening disorder caused by exposure to heparin and characterized by high morbidity and mortality. It is largely underestimated because of its heterogeneous presentation, ranging from a decrease in platelet count antibody positivity to serious thrombotic complications. Haemodialysis (HD) patients represent a high-risk population due to anticoagulation use during extracorporeal treatments. It usually occurs in the first weeks after the start of HD, although it has been reported in chronic HD patients after surgery procedures. When the diagnosis of HIT is formulated, heparin must be promptly stopped and an alternative anticoagulant has to be started. The aim of this review is to provide a comprehensive overview on the pathogenesis, diagnosis and treatment of HIT in HD in order to increase the awareness of physicians of this important clinical syndrome to promptly start the specific treatment.

## INTRODUCTION

Heparin-induced thrombocytopenia (HIT) is a serious complication related to heparin exposure, estimated to occur in 1/5000 hospitalized patients [1, 2]. Patients with HIT are at risk of developing thrombosis (30–50%), the incidence of which reaches 50–89% in untreated patients. Even with early recognition and intervention, morbidity and mortality are 7.4% and 1.1%, respectively [3].

The discontinuation of heparin alone is not sufficient to prevent thrombotic complications [2, 3].

Haemodialysis-related HIT (HD-HIT) usually occurs in the first weeks after HD initiation, but it may also appear in chronic HD, particularly during inflammatory/perioperative states [4]. Since unfractionated heparin (UFH) and low molecular weight heparin (LMWH) are routinely used to avoid blood coagulation during HD, patients are continuously exposed to heparin. HIT can occur according to the type of heparin, dose and duration of treatment; women, older age and surgical patients are at higher risk [1, 5].

Here we review the pathophysiology, diagnosis and current standards of treatment and discuss the outcome prediction and management of HD-HIT.

## THROMBOCYTOPENIA IN HD

**Figure 1:**
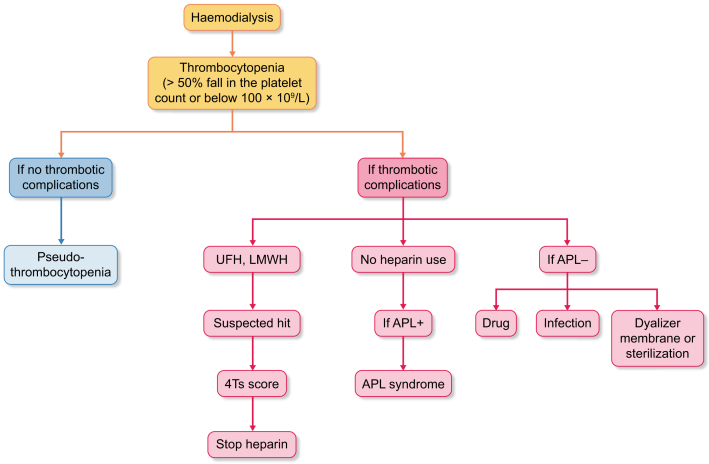
Flow chart of diagnosis and treatment algorithm.

Patients affected by kidney failure (KF) are considered at high risk to develop thrombocytopenia. There are multiple causes of thrombocytopenia in HD patients and the differential diagnosis is essential: 1) pseudo-thrombocytopenia (PTCP), related to ethylenediaminetetraacetic acid (EDTA) (0.1–2%) [6, 7], can be associated with tumours, infections, autoimmune diseases, liver diseases, low temperature exposure and sometimes aspirin and warfarin [8]; 2) dialyser-related thrombocytopenia, mainly due to membrane bio-incompatibility and the sterilization method of dialyser membranes [9, 10]—in particular, older membranes increase complement activation, which triggers leucocytosis, leucocytes sequestration in the lungs and platelet activation, but fortunately new-generation membranes are more biocompatible than the older ones and reduce the risk of the above-mentioned phenomena [11]; 3) antiphospholipid syndrome (APS) secondary to autoimmune disorders such as lupus erythematosus; 4) infection-induced thrombocytopenia, associated with repeated arteriovenous fistula punctures and the use of central venous catheters; and 5) drug-induced thrombocytopenia (DITP), which includes not only a variety of anti-infective agents, such as sulphonamides, quinolones, vancomycin, trimethoprim/sulfamethoxazole, linezolid and piperacillin/tazobactam, but also heparin and, even if rarely used in HD, glycoprotein IIb/IIIa inhibitors (such as abciximab, tirofiban and eptifibatide) (Fig. 1).

## HIT

**Figure 2:**
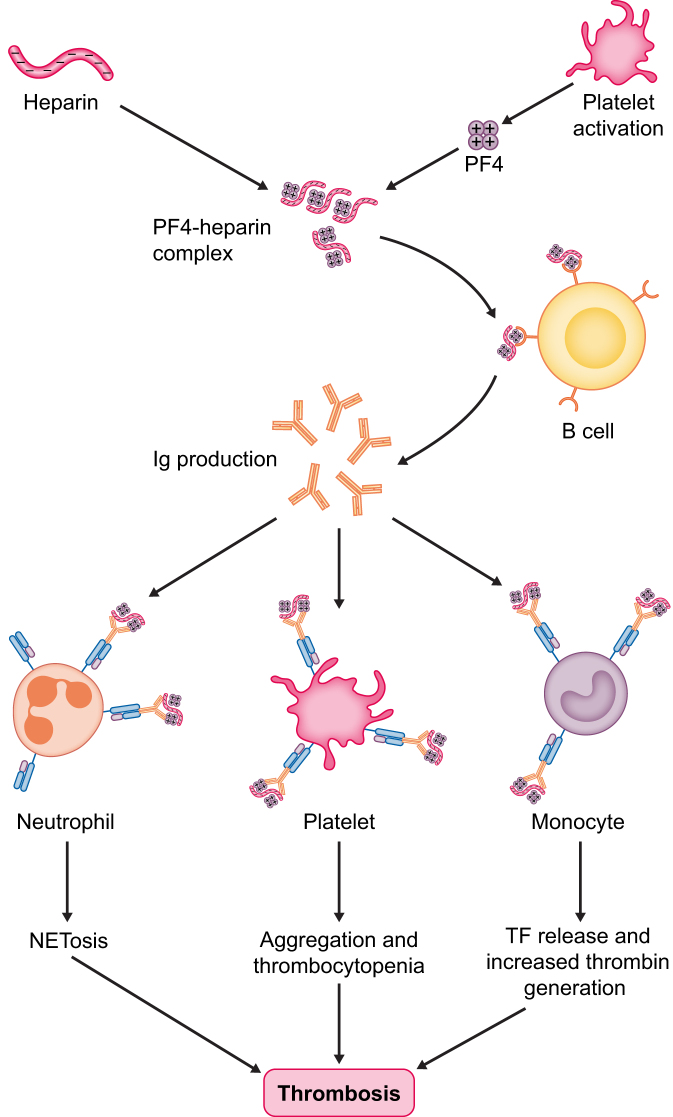
HIT pathogenesis. Exposure to heparin along with the liberation of platelet factor-4 (PF4) by activated platelets leads to the formation of heparin/PF4 complexes, which stimulates B cells to produce specific antibodies. The antibodies (and in particular IgG) bind the FcRγIIa on the surface of neutrophils leading to NETosis, monocytes leading to tissue factor (TF) release and increased thrombin generation, platelets causing aggregation and platelet consumption. The activation of these pathways contributes to thrombosis development.

Two types of HIT exist. Type 1 (10%) is a benign, non-immune-related thrombocytopenia due to direct interaction between heparin and platelets. It usually presents within the first 48–72 hours of drug initiation and is associated with mild thrombocytopenia (rarely <100 000/mm^3^), which often resolves after heparin withdrawal. Type 2 is a more severe immune-mediated thrombocytopenia. It usually occurs within 5–14 days from exposure but can start abruptly in patients who had exposure to heparin in the previous 90 days [12]. HIT is caused by the electrostatic interaction between heparin, negatively charged, and PF4, a positively charged chemokine released from alpha granules of activated platelets; immune cells recognize the PF4/heparin complex and produce immunoglobulin G (IgG). The anti-PF4/H IgG binds Fc receptors expressed on platelets (FcRgIIa), leading to their activation, thrombosis and consumption thrombocytopenia. More recently, interaction of the immune complexes with neutrophil FcγRIIA receptors and subsequent neutrophil extracellular trap-osis (NETosis) has also been shown to be a major contributing factor in HIT-associated thrombosis (Fig. 2) [13]. IgM and IgA rarely cause HIT, and not all IgG causes HIT, but only a subset of them recognizing ultra-large H-PF4 complexes, which are highly immunogenic.

Based on onset time, HIT is classified as acute, if diagnosed within the last month from heparin exposure; subacute HIT, diagnosed after 1–3 months, and further classified as subacute A, with a normal platelet count and positive immunoassays, and subacute B, with a negative functional assay; and remote HIT, an old HIT of >3 months, with rarely detectable H-PF4 antibodies [12, 14–18].

Risk factors for HIT include prolonged heparin exposure; use of UFH compared with LMWH (3% versus 1.5%, respectively) [19, 20]; cardiac and orthopaedic surgery (risk of 1% and 5%); cardiovascular disease, patients in critical care units, HD (from 0.3 to 3.2%); and female gender, with double risk compared with men, likely due to increased immune response [5].

## HD-HIT

HD-HIT is suspected when the platelet count decreases 30% or <150 × 10^9^ cells/l, a criterion that is less strict compared with the general population (>50% decrease in the platelet count or <100 × 10^9^ cells/l) [21]. It usually occurs in the first weeks after the start of HD, although it has been reported in patients undergoing HD for longer periods of time. HD patients are considered a risk group, and it was recently found that up to 12% develop HIT [4, 21]. In fact, dialyser membranes and circuit lines are procoagulant factors and consequently UFH and LMWH are commonly used to avoid blood coagulation during HD, thus routinely exposing patients to heparin [1, 5].

The frequency of HIT varies widely because assays used to detect HIT antibodies have heterogeneous specificity and sensitivity. Previous data reported an estimated incidence of HIT in the general HD population of 0.6%, whereas in newly treated patients it is 3.9% [22, 23]. Most episodes of HD-HIT occur in the first 2 weeks of HD, especially in acute inflammatory/perioperative settings.

The prevalence of HIT in KF patients is not well established. Recently Kaur *et al*. [24] reported a HIT prevalence rate of 4.6% in KF patients who received UFH during hospitalization for various medical conditions. This incidence is dramatically higher compared with the general population who experienced HIT during hospitalization (1/5000), and renal failure was found as the second most important risk factor for HIT among various procoagulant conditions after congestive heart failure.

## DIAGNOSIS

Thrombocytopenia occurs in up to 55% of patients with KF, making the differential diagnosis pivotal. Of note, during each HD session a mild and transient reduction in platelet count (up to 15%) can occur due to platelet adhesion and complement activation [25]. HD-HIT should be suspected when the platelet count persistently decreases following heparin exposure.

It can usually occur with unexpected clotting in the circuit and abrupt fistula thrombosis despite an optimal dose of heparin and in a time ranging from 7 to 30 days from the first heparin exposure [26]. HD-HIT should be suspected in patients with thrombocytopenia in the time window for HIT even if circuit clotting is absent, after excluding other causes of clotting such as slow blood flows, high haematocrit, high ultrafiltration rate, intradialytic blood and blood product transfusion and intradialytic lipid transfusion [23]. Other clinical manifestations are deep vein thrombosis (in ≈50%), with a 10–25% risk of pulmonary embolism; ischaemic necrosis of the limbs (5–10%), with an amputation rate of 1–3%; acute myocardial infarction (≈3%); stroke (≈4%) and, rarely, mesenteric ischaemia [22]. Mortality associated with HIT ranges from 5 to 30%.

**Figure 3:**
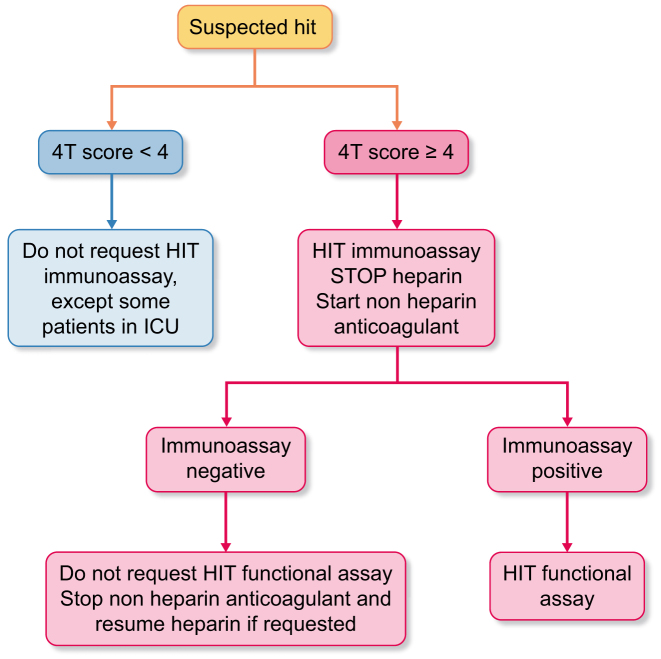
Algorithm for diagnosis of HD-HIT.

Diagnostic algorithms combine clinical and laboratory tests for HIT diagnosis and imaging can be used to evaluate thrombosis. The 4Ts is the preferred scoring method (Table 1). The HIT Expert Probability (HEP) score is an alternative in non-dialysis patients, although it is time-consuming and has controversial accuracy. A low 4Ts score excludes HIT and laboratory testing is not recommended, with some exceptions: patients in intensive care unit (ICU) [British Society for Haematology (BSH) grade 2B], those receiving extracorporeal membrane oxygenation (BSH grade 2C) [16] and when the pretest probability is uncertain due to missing or unclear data [14, 17]. If the risk of HIT is moderate–high (4Ts score ≥4), guidelines recommend stopping heparin, starting an alternative anticoagulant therapy and confirming the diagnosis with a combination of immunological and functional tests (Fig. 3). The most common immunological assay is enzymatic immunoassay (EIA), with a negative predictive value of 98% for detecting H-PF4 antibodies, but variable specificity [27]. In KF the presence of H-PF4 antibodies did not correlate with the incidence of HIT [28]. Beyond EIA, there are other available immunoassays that present different specificity and sensibility [i.e. particle gel immunoassay, IgG-specific chemiluminescence, lateral flow immunoassay and latex immunoturbidimetric assay). Positive EIA requires confirmation by a functional test in order to define if H-PF4 antibodies are pathogenic. The most common is the platelet serotonin-release assay (SRA), which is considered the gold standard because it is characterized by high sensitivity and specificity for HIT (≈95% each), but it is not used in routine practice [29]. Another functional test is the heparin-induced platelet activation assay [30], which is faster and easier to be perform. It recently showed good positive agreement with the SRA for HIT diagnosis [31, 32].

**Table 1:** The 4Ts score.

Parameter	0 point	1 point	2 points
Thrombocytopenia	Platelet decrease ˂30% or platelet nadir ˂10 × 10^9^/l	Platelet decrease >30–50% or platelet nadir 10–19 × 10^9^/l	Platelet decrease >50% and platelet nadir ≥20 × 10^9^/l
Timing of platelet count decrease	≤4 days without recent exposure	Likely 5–10 days but not definite, after 10 days, or ≤1 day + previous heparin exposure 30–100 days ago	5–10 days or ≤1 day + previous heparin exposure within 30 days
Thrombosis or other sequelae	None	Progressive or recurrent thrombosis, suspected thrombosis or non-necrotizing (erythematous) skin lesions	Confirmed new thrombosis, skin necrosis, acute systemic reaction after intravenous bolus heparin
Other causes of thrombocytopenia	Definite	Possible	None

Score ≥6 points: high probability of HIT (64%); score 4–5 points: intermediate probability of HIT (14%); score 0–3 points: low probability of HIT (˂5%). A score ranging from 0 to 3 points corresponds to a low probability of HIT (inferior to 5%),; from 4 to 5 the probability is moderate (14%) and from 6 to 8 points, the probability is high (64%).

If the 4Ts score is high and EIA is strongly positive, a functional assay is not necessary to confirm a HIT diagnosis. It should be noted that the predictive value of EIA has not been validated in specific HD populations, so functional assays in these patients acquire a pivotal role in the HIT diagnosis.

## MANAGEMENT

When HD-HIT is suspected the optimal management consists of promptly discontinuing dialysis and replacing the whole extracorporeal circuit (ECC); restarting dialysis without heparin; verifying that there are no clots in the circuit; using heparin-free dialysis (consider the use of flushes of saline solution, pre-dilution haemodiafiltration, high biocompatibility filters as antithrombogenic hydrophilic dialysis membrane [33, 34] and citrate anticoagulation dialysis); closing central venous catheters (CVCs) with tissue plasminogen activator, urokinase or trisodium citrate and non-heparin anticoagulant; searching for asymptomatic deep venous thrombosis; and considering a switch to peritoneal dialysis in HD patients with active bleeding or high bleeding risk. The following should be avoided: heparin flushing on non-HD days; heparin-coated CVCs; heparin-grafted dialysis membranes; heparin in both the filling circuit lines and in the CVC closing lock; LMWH, due to the possibility of cross-reactivity following the induction with UFH antibodies; and platelet transfusion and the insertion of a vena cava filter, as they could induce or exacerbate thromboembolic complications [33].

For patients requiring renal replacement therapy (RRT), the American College of Chest Physicians (ACCP) suggests argatroban or danaparoid (grade 2C) (Table 2), while for patients with prior HIT requiring ongoing RRT or catheter locking, regional citrate is preferred over heparin or LMWH (grade 2C). The American Society of Haematology (ASH) similarly suggests argatroban, danaparoid or bivalirudin for acute HIT and citrate for subacute or remote HIT when anticoagulation is required only to maintain circuit patency. The BSH suggests argatroban or danaparoid over citrate anticoagulation in active HIT (grade 2B) and argatroban, danaparoid or citrate for patients with previous HIT (grade 1C). Specific danaparoid regimens can be used to maintain ECC patency while avoiding drug accumulation (Table 2) [36–39]. No direct comparative trials exist on non-heparin anticoagulants during RRT in HIT.

**Table 2:** Danaparoid treatment regimens in dialysis.

Current HIT and CRRT in the absence of clotting in non-HIT or HIT patients	i.v. loading bolus as follows:2250 U i.v. for patients 55–90 kg body weight, 1500 U i.v. for patients <55 kg body weight, 3750 U i.v. for patients >90 kg body weight, plus in all patients an immediate ‘step-down’ i.v. infusion of 400 U/h for 4 h, then 300 U/h for 4 h, then 150–200 U/h maintenance infusion for 7 days, or longer if required.In patients with an estimated glomerular filtration rate <30 ml/min/1.73 m^2^ and/or on CRRT the maintenance dose should be reduced to 150 U/h.The maintenance infusion rate can be adjusted according to the risk of bleeding.If monitored at steady state within 6 h after the start of treatment the plasma anti-Xa activity level should be between 0.4 and 0.8 U/ml.If the plasma anti-Xa level is outside this range or, in the absence of monitoring, thrombosis or bleeding occur, then the maintenance infusion rate can be adapted accordingly by an increase or decrease of 20% of the maintenance infusion rate.
CRRT use in case of clotting of the circuit in non-HIT and HIT patients	2250 U i.v. bolus plus step-down i.v. infusion of 400–600 U/h for 4 h, then 300 U/h for 4 h, then 100–400 U/h (maintenance rate)
Intermittent HD in non-HIT and HIT patientsHD not daily	If plasma anti-Xa monitoring is available:Bolus pre-dialysis for the first two dialyses: 2250 U i.v. if patient is <55 kg and 3750 U i.v. if patient is ≥55 kg.For the third and subsequent dialyses, the pre-dialysis dose is dependent upon the pre-dialysis anti-Xa activity of the previous dialysis:<0.3 U/ml: 3000 U i.v. pre-dialysis (<55 kg: 2000 U)0.3–0.35 U/ml: 2250 U (<55 kg: 1500 U)0.35–0.4 U/ml: 2000 U (<55 kg: 1500 U)>0.4 U/ml: danaparoid not required for that dialysis, but the pre-dialysis plasma anti-Xa activity level will determine the dose required for the following dialysis
HD daily	As above, but 2250 U (<55 kg: 2000 U) prior to the second dialysis.By the fourth to sixth dialysis the dose will have reached a steady state and will be the same for subsequent dialyses; however, it is advised to monitor for bleeding or circuit clotting/systemic thrombosis. If there is a significant risk of systemic thrombosis or a thrombosis is evident, then use the continuous infusion of regimen 2a instead. However, since renal failure may lead to accumulation, the maintenance infusion rate should be 150 U/h, as described above, to avoid plasma anti-Xa activities >0.8 U/ml.
Flush doses for all patients	750 U danaparoid diluted into 50 ml saline. Use 5–10 ml of this solution to flush intravascular lines/access ports as required.

CRRT: continuous renal replacement therapy; i.v.: intravenous.

Specifically, even in patients with remote HIT who are on RRT and require anticoagulation to prevent thrombosis of the dialysis circuit, the ASH guideline panel suggests regional citrate rather than heparin or other non-heparin anticoagulants. A danaparoid-based protocol is used in HD patients with a history of HIT (Table 2).

Limited data are available on prolonged heparin exposure and postoperative HIT in chronic HD. Surgical procedures can release PF4 and postoperative thrombocytosis can mask thrombocytopenia, making platelet monitoring after day 5 crucial for early HIT detection [21].

## TREATMENTS

The current management of HD-HIT incorporates a stepwise approach involving multiple strategies (Fig. 1, Table 1).

**Figure 4:**
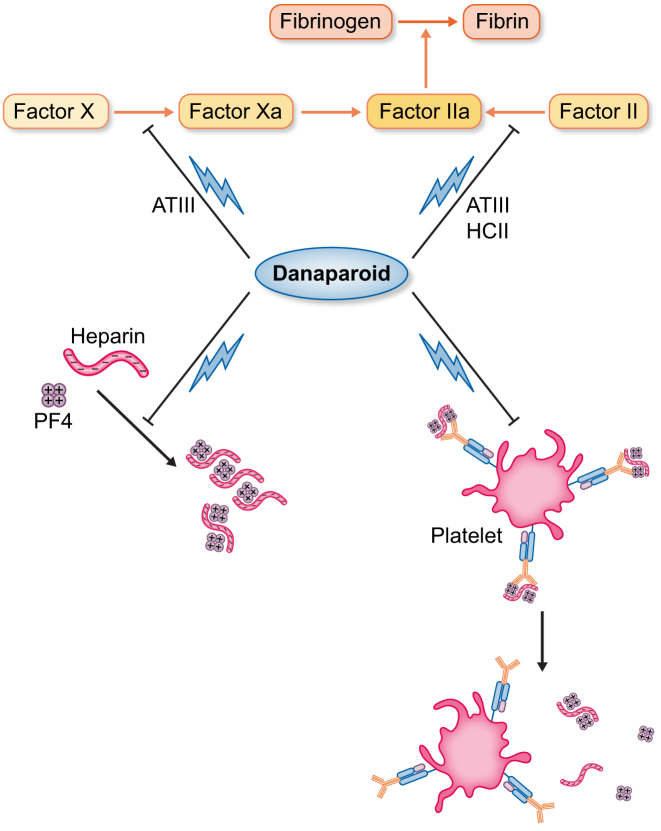
Mechanism of action of danaparoid. Danaparoid exerts its activity through the inhibition of factor Xa and thrombin (factor IIa) generation, thus preventing thrombus formation and thrombin-induced platelet aggregation. Danaparoid also acts by inhibiting the formation of new heparin/PF4 complexes and disrupting the circulating ones and those attached to the platelet surface, thus reducing the immune response and decreasing platelet activation.

1. Stop heparin. All heparin sources must be immediately discontinued if HIT is suspected or confirmed. However, thrombotic risk remains at up to 52% for 30 days and decreases only after 3 months, requiring prompt initiation of a non-heparin anticoagulant [40].
2. Initiation of a non-heparin anticoagulant. Parenteral non-heparin anticoagulants at a therapeutic dosage remain the cornerstone of acute HIT treatment in patients with suspected HIT and high-probability 4T score as recommended by the ASH. For intermediate probability 4T score, prophylactic or therapeutic doses depend on bleeding risk; for low-probability cases, immunoassay is unnecessary, except patients in the ICU. Approved agents include danaparoid (EU/Canada/Australia/New Zealand/Japan) and argatroban (USA/Canada/EU/Japan, South Korea) and bivalirudin (USA only).
  - Argatroban. Argatroban is a direct thrombin inhibitor (DTI) that is predominantly metabolized in the liver by CYP3A4 oxidases and excreted in faeces [41]. It has a short half-life (40–50 min), so it may be favoured in case of high bleeding risk or potential surgery [14, 42]. It is administered as a continuous intravenous infusion. It may be used during percutaneous coronary intervention (PCI) when bivalirudin is unavailable and in pregnancy. Argatroban can safely be used in HD without dose adjustment since clearance via high-flux membranes is negligible (Table 3) [43, 44].
  - Danaparoid. Danaparoid sodium is a non-heparin glycosaminoglycan mixture developed as an alternative to heparin in 1977 and approved in 1994 for the treatment of acute and remote HIT. Danaparoid is the only drug to have a significant impact on HIT pathogenesis, through two mechanisms: it acts on the coagulation cascade, inhibiting thrombin-induced platelet aggregation, thrombin activation and thrombus formation through indirect (via antithrombin activation) inhibition of factor X activation, and by direct inhibition of thrombin activation of factor IX. Moreover, it exerts an immunomodulation action by decreasing PF4 binding to platelets and displacing PF4-heparin complexes from platelet surfaces (Fig. 4) [45]. Danaparoid is mainly metabolized by the kidneys, requiring dose adjustments in KF. Its half-life is ≈25 hours and 2 hours for anti-Xa and anti-IIa activity, respectively, and 7 hours for thrombin generation inhibition. Danaparoid has no direct antidote and heparin antidotes, such as protamine sulphate, are not effective, so it may not be suitable for patients requiring emergency surgery, cardiac surgery or with a high risk of bleeding. Plasmapheresis can remove danaparoid from the circulation and fresh frozen plasma transfusions may decrease its effect [46]. It is usually administered as a continuous intravenous infusion after a loading dose bolus according to the patient’s body weight (Table 2). In the switch phase and in patients who need prophylactic anticoagulation until 3 months from an acute HIT, it can be administered subcutaneously, with an absolute bioavailability of nearly 100%.
    Anti-Xa activity plasma monitoring to measure danaparoid activity (therapeutic target range of 0.5–0.8 activity/ml) is not readily available in many labs but generally is not necessary unless under specific conditions: adults with a body weight <55 kg or >90 kg, patients with clinically significant renal impairment and the paediatric population.
    After the recommended intravenous loading dose and continuous infusion rates for at least 1–2 weeks, steady-state anti-Xa activity levels are reached, so blood samples for monitoring can be taken at any time. Instead, after therapeutic subcutaneous doses, monitoring of anti-Xa activity levels should be performed 3–4 hours after injection.
    Limitations on the use of danaparoid include hypersensitivity, recent haemorrhagic stroke, severe and uncontrolled hypertension, active gastroduodenal ulcer, diabetic retinopathy and spinal or epidural anaesthesia or loco-regional anaesthesia when danaparoid sodium has been used in the previous 24 hours.
    Some contraindications do not apply if the patient has HIT and no alternative antithrombotic treatment is available, such as severe haemorrhagic diathesis (e.g. haemophilia and idiopathic thrombocytopenic purpura), severe renal and hepatic insufficiency, acute bacterial endocarditis, recent (<1 week) or active bleeding (e.g. intracranial, gastrointestinal, intraocular or pulmonary), damage to the central nervous system or spinal or ophthalmological surgery.
  - Bivalirudin. Bivalirudin is a DTI that is mainly metabolized by proteolytic cleavage (80%) and partially by renal excretion (20%) and has a short half-life (25 min). It is US Food and Drug Administration approved for patients with current or previous HIT during PCI. ACCP guidelines recommend the use of bivalirudin for patients with acute or subacute HIT who require urgent cardiac surgery. It is administered as a continuous intravenous infusion and a dose reduction is used in CKD patients and HD patients (Table 3) [15, 47].
3. Oral anticoagulants. Warfarin is contraindicated in acute HIT because it reduces protein C and S levels, increasing the generation of thrombin and leading to a higher risk of thromboembolic complications. For patients already receiving warfarin, reversal with vitamin K is suggested during the acute phase of HIT.
4. Transition to oral anticoagulant therapy. Transition to oral anticoagulants should begin after recovery of the platelet count. Vitamin K antagonists (VKAs) should be started without loading doses and overlapped with a parenteral anticoagulant for at least 5 days until the International Normalized Ratio (INR) reaches the target. The transition from argatroban to VKAs requires the discontinuation of argatroban when the INR is >4, leaving only VKA, then repetition of the INR after 4–6 hours and the use of argatroban if the INR is <2 until the INR reaches the value of 2 [14].
5. Duration of anticoagulant treatment. In HIT with thrombosis, 3 months of anticoagulation is recommended (BSH grade 1A) [16]. For HIT without thrombosis, ASH suggests anticoagulation until platelet count recovery [14]. The ACCP suggests 4 weeks of anticoagulation, whereas the BSH recommends anticoagulation until platelet count recovery or for at least 4 weeks [48]. Thrombosis Canada recommends at least 4 weeks of anticoagulation and its continuation until platelet recovery [17].
6. Fondaparinux is a synthetic pentasaccharide that acts as an anticoagulant, inhibiting factor Xa. The available data concerning its use in HIT treatment are controversial. In fact, its utilization in HIT is described only in one small prospective study and in one small retrospective study [49], while some case reports described fondaparinux-related HIT [50], HIT exacerbation and unsuccessful prophylactic use for delayed HIT [51]. Fondaparinux is nearly completely dependent on renal clearance, thus it is contraindicated in patients with renal insufficiency (creatinine clearance <30 ml/min), while in HD it is only slightly dialyzable (20%). Indeed, on the basis of the scarce quality evidence so far available, it is currently not approved as treatment in HD-HIT [2].
7. Sulodexide, a widely available and low-cost anticoagulant, could be an interesting alternative in HD-HIT, but only one case report has been described [52] and only one comparative study showed a stronger anticoagulation effect of sulodexide than enoxaparin [53].

**Table 3:** Non-heparin anticoagulation in patients with acute HD-HIT.

	Danaparoid	Argatroban	Bivalirudin
Mechanism of action	1. Inhibition of factor X and thrombin activation of factor IX2. Inhibition of formation and disruption of PF4/heparin complexes and their binding to platelets	Direct inhibition of thrombin	Direct inhibition of thrombin
Metabolism	Kidney	Liver	Circulating proteases (80%), kidney (20%)
Half-life	25 h (Xa)	40–50 min	25 min
dosage	Bolus i.v.:• <60 kg: 1500 U• 60–75 kg: 2250 U• 75–90 kg: 3000 U• >90 kg: 3750 UAccelerated initial infusion: 400 U/h for 4 h, then 300 U/h for 4 hMaintenance infusion:• Normal renal function: 200 U/h• Renal dysfunction: 150 U/h	Bolus: noneMaintenance:• Normal organ function: 2 μg/kg/min• Hepatic dysfunction (bilirubin >1.5 mg/dl): 0.5–1.2 μg/kg/min• Anasarca, heart failure, post-cardiac surgery: 0.5–1.2 μg/kg/min	Bolus: noneMaintenance:• Normal organ function: 0.15 mg/kg/h• Renal or liver dysfunction: dose reduction may be required
Serum parameters to control	Anti-Xa (danaparoid-specific): 0.5–0.8 μg/ml	APTT: 1.5–3 times baseline value	APTT: 1.5–2.5 times baseline
Use	• Liver dysfunction• Pregnancy• HD	• PCI• CrCl <30 ml/min• HD• Pregnancy	• PCI• HD• Liver dysfunction
Avoid	• Possible *in vivo* cross-reactivitywith HITantibodies• Urgent surgery• Cardiac surgery• High risk of bleeding	• Prolongs PT; caution if transitioning to warfarin• Child–Pugh class B and C liver dysfunction: avoid or reduce dose• High cost	• Prolongs PT; caution if transitioning to warfarin• High cost

APTT: activated partial thromboplastin time; CrCl: creatinine clearance; PT: prothrombin time.

## OUTCOME PREDICTION

Monitoring of the platelet count during treatment is crucial. It should be monitored daily during week 1, on alternate days during weeks 2–3, then weekly or monthly. The expected recovery in platelet levels starts after 48 hours, reaching normal values after 4–7 days and after 2 weeks if the thrombocytopenia is severe or in case of disseminated intravascular coagulation [54]. The development of danaparoid clinical cross-reactivity (defined as a new or persistent platelet count reduction and/or a new or extended thrombosis) may worsen patient outcome. In this case, danaparoid should be discontinued and an alternative treatment should be considered [55].

## CONCLUSIONS

Our work aimed to provide an overview of HIT, which is an overlooked but severe condition that can occur in many settings and, in particular, in patients undergoing HD. We tried to give readers some tips to suspect and promptly identify this disease along with some suggestions to efficiently manage it. HD patients are at high risk to develop HD-HIT, which is underestimated but fatally associated with high morbidity and mortality. Thus both diagnosis and treatment of HD-HIT still represents a challenge for clinicians. We consider it of critical importance to increase physician awareness of this potentially fatal diagnosis in order to promptly withdraw heparin treatment and start specific therapy.

## Data Availability

The data are provided in the article and references.
